# High Yield of Wax Ester Synthesized from Cetyl Alcohol and Octanoic Acid by Lipozyme RMIM and Novozym 435

**DOI:** 10.3390/ijms130911694

**Published:** 2012-09-17

**Authors:** Chia-Hung Kuo, Hsin-Hung Chen, Jiann-Hwa Chen, Yung-Chuan Liu, Chwen-Jen Shieh

**Affiliations:** 1 Biotechnology Center, National Chung Hsing University, 250 Kuo-kuang Road, Taichung 402, Taiwan; E-Mail: jahomekuo@gmail.com; 2 Department and Graduate Program of Bioindustry Technology, Dayeh University, 168 University Road, Chang-Hwa, 515, Taiwan; E-Mail: cjshieh@nchu.edu.tw; 3 Graduate Institute of Molecular Biology, National Chung Hsing University, 250 Kuo-kuang Road, Taichung, 402, Taiwan; E-Mail: jhchen@dragon.nchu.edu.tw; 4 Department of Chemical Engineering, National Chung Hsing University, 250 Kuo-kuang Road, Taichung, 402, Taiwan; E-Mail: ycliu@dragon.nchu.edu.tw

**Keywords:** optimization, wax esters, lipase, esterification, *Rhizomucor miehei*, *Candida antarctica*

## Abstract

Wax esters are long-chain esters that have been widely applied in premium lubricants, parting agents, antifoaming agents and cosmetics. In this study, the biocatalytic preparation of a specific wax ester, cetyl octanoate, is performed in *n*-hexane using two commercial immobilized lipases, *i.e.*, Lipozyme^®^ RMIM (*Rhizomucor miehei*) and Novozym^®^ 435 (*Candida antarctica*). Response surface methodology (RSM) and 5-level-4-factor central composite rotatable design (CCRD) are employed to evaluate the effects of reaction time (1–5 h), reaction temperature (45–65 °C), substrate molar ratio (1–3:1), and enzyme amount (10%–50%) on the yield of cetyl octanoate. Using RSM to optimize the reaction, the maximum yields reached 94% and 98% using Lipozyme^®^ RMIM and Novozym^®^ 435, respectively. The optimum conditions for synthesis of cetyl octanoate by both lipases are established and compared. Novozym^®^ 435 proves to be a more efficient biocatalyst than Lipozyme^®^ RMIM.

## 1. Introduction

Wax esters are long-chain esters with chain lengths of 12 or more. They can be extracted from a variety of natural sources, including honeycomb, jojoba seeds, carnauba, sperm whale, skin lipids, sheep wool and seafowl feathers. Wax esters are of industrial importance. They are used as additives or auxiliaries in lubricants, polishes, plasticizers and coating materials [1]. For example, cetyl octanoate, a wax ester of 24 carbons, is one of the main wax esters in seafowl feathers to protect birds from becoming wet and make the feathers flexible. Industrially, cetyl octanoate has been widely used as an oil base in cosmetics (cleansers, conditioners, and moisturizers) due to its excellent moisture retaining ability and non-greasy feeling on the skin.

Traditionally, surface wax esters are extracted from their natural sources by a short solvent wash [2] followed by several expensive purification procedures [3]. With the steadily growing demand for wax esters in the food, pharmaceutical and cosmetic industries, methods for chemical or enzymatic synthesis of wax esters have been developed. Ieda *et al*. [4] reported the synthesis of wax esters from long-chain fatty acids and alcohols by chemical reactions. Hadzir *et al.* [5] reported the synthesis of oleyl oleate through lipase-catalyzed alcoholysis of triolein with oleyl alcohol. Salis *et al.* [6] reported the synthesis of wax esters (cetyl myristate, cetyl palmitate, cetyl oleate and cetyl stearate) through lipase-catalyzed alcoholysis of sheep milk fat with cetyl alcohol. Among the many synthesis methods reported, lipase-catalyzed synthesis of wax esters has drawn much attention. Lipase catalyzes hydrolysis of triacylglycerol into glycerol and fatty acids in aqueous solution [7]. In organic solvents, the lipase can catalyze the esterification [8,9], alcoholysis [10], acidolysis or interesterification to produce esters or structured lipids [11]. Lipase-catalyzed reactions usually take place at atmospheric pressure, moderate pH and moderate temperature, which is considered an effective way to overcome the drawbacks of chemical processes. As free lipase is generally insoluble or even denatured in organic solvents, immobilized lipases are nowadays widely used to ensure that stability and activity of lipase can be retained and the problem of substrate inhibition may be overcome. The lipases used most often are lipases from *Rhizomucor miehei* [12] or *Candida antarctica* [13]. These two lipases can also catalyze the synthesis of wax esters in supercritical carbon dioxide [14–16].

Generally, lipase-catalyzed esterification reactions are affected by several parameters, such as species of lipase, reaction solvent, reaction time, synthesis temperature, water activity and acyl donors [17]. For the optimal synthesis of wax esters, a model for the optimization of the most relevant operational parameters is required. Compared with a one-factor-at-a-time design, which is adopted most frequently in the literature, the fractional factorial experimental design is more efficient in reducing the number of experimental runs and time for investigating the optimal conditions of wax esters synthesis. To date, response surface methodology (RSM) has been applied successfully for modeling various biochemical processes, such as enzyme immobilization [18,19], extraction [20,21], fermentation [22] and biofuel production [23].

In this work, the lipase-catalyzed synthesis of cetyl octanoate was investigated. Lipases from *Rhizomucor miehei* (Lipozyme^®^ RMIM) and from *Candida antarctica* (Novozym^®^ 435) that are immobilized on a macroporous anionic resin and an acrylic resin, respectively, were used as biocatalysts. RSM, using a 5-level-4-factor central composite rotatable design (CCRD), was employed to investigate the relationships between the reaction variables (reaction time, reaction temperature, substrate molar ratio, and enzyme amount) and response (yield %) in order to obtain the optimal condition for lipase-catalyzed synthesis of wax esters.

## 2. Results and Discussion

### 2.1. Model Fitting

RSM is a useful statistical technique for investigating complex processes. By this technique, optimal conditions for the process can be obtained in the laboratory. A scale-up procedure may then be employed for industrial production. A 5-level-4-factor CCRD with 27 runs was carried out in order to determine the main factors for the synthesis of cetyl octanoate using Lipozyme^®^ RMIM and Novozym^®^ 435. The yields obtained from two enzymes are listed in Table 1. For Lipozyme^®^ RMIM, the greatest yield (94.86%) was treatment 7 (4 h, 60 °C, molar ratio 2.5:1, and 40% enzyme), and the smallest yield (only 44.62%) was treatment 13 (1 h, 55 °C, molar ratio 2:1, and 30% enzyme). For Novozym^®^ 435, the greatest yield (97.95%) was treatment 26 (4 h, 50 °C, molar ratio 2.5:1, and 40% enzyme), and the smallest yield (64.16%) was treatment 10 (2 h, 50 °C, molar ratio 1.5:1, and 20% enzyme). It was noticed that the high yields of cetyl octanoate (>90%) for both enzymes were obtained at high levels of enzyme amount (≥40% (*w*/*w*)), e.g., treatments 3, 7, 22 and 26. The results suggested that the external mass transfer limitation was essentially eliminated at a high enzyme amount condition, which is in agreement with previous studies [24,25].

From the SAS software (SAS Institute, Cary, NC, USA) output of RSREG, the second-order polynomial equations for synthesis yields using respectively Lipozyme® RMIM and Novozym® 435 are given below:

Lipozyme^®^ RMIM (Y_1_):

(1) $$\begin{array}{l} Y_{1}\;(\%)=-21.090+22.743X_{1}+0.529X_{2}+18.058X_{3}+0.165X_{4}-2.474{X_{1}}^{2}+\\ 0.096X_{1}X_{2}-0.004{X_{2}}^{2}-0.281X_{1}X_{3}+0.066\;X_{2}X_{3}-6.101{X_{3}}^{2}-0.088X_{1}X_{4}-\\ 0.004X_{2}X_{4}+0.149X_{3}X_{4}+0.008{X_{4}}^{2} \end{array}$$

Novozym^®^ 435 (Y_2_):

(2) $$\begin{array}{l} Y_{2}\;(\%)=-161.291+39.949X_{1}+1.479X_{2}+44.877X_{3}+4.831X_{4}-1.800{X_{1}}^{2}-\\ 0.230X_{1}X_{2}+0.010{X_{2}}^{2}-1.614X_{1}X_{3}-0.207\;X_{2}X_{3}-4.210{X_{3}}^{2}-0.259X_{1}X_{4}-\\ 0.033X_{2}X_{4}-0.161X_{3}X_{4}-0.023{X_{4}}^{2} \end{array}$$

**Figure 1:**
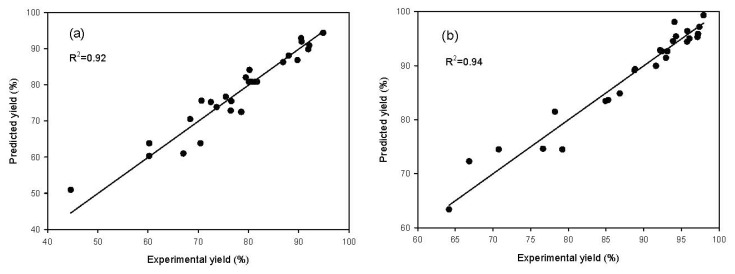
The relationship between predicted and experimental yield of cetyl octanoate for the two lipases tested: (**a**) Lipozyme^®^ RMIM and (**b**) Novozym^®^ 435.

The plots of experimental values of the yield (%) *versus* those calculated from Equations 1 and 2 are shown in Figure 1. The predicted values from RSM models fit well with the experimental values with a high determination coefficient of 0.92 and 0.94 respectively for Lipozyme^®^ RMIM and Novozym^®^ 435.

The analysis of variance (ANOVA) from Table 2 indicated that the second-order polynomial model (Equations 1 and 2) was statistically significant and adequate to represent the actual relationship between the responses and the variables, with a very small *p*-value (<0.0001) and a satisfactory coefficient of determination (*R*^2^ = 0.92~0.94). The ANOVA results also indicated that the linear terms had a very significant (*p* < 0.0001) influence on the yield for both models. However, the effects of quadratic and cross product terms were only significant for Novozym^®^ 435. Furthermore, the overall effect of the four manipulated variables on the yield of cetyl octanoate was analyzed by a joint test (Table 3). The results indicated that the reaction time and enzyme amount were important factors for Lipozyme® RMIM. Basri *et al*. [26] reported that the reaction time and enzyme amount were significant variables for lipase-catalyzed synthesis of palm-based wax ester using lipase from *Rhizomucor miehei*. On the other hand, the reaction time, reaction temperature, molar ratio and enzyme amount were all important factors for Novozym^®^ 435. These factors exerted a statistically significant overall effect (*p* < 0.05) on the cetyl octanoate production.

### 2.2. Mutual Effect of Parameters

Using surface response plots of the quadric polynomial model, the relationships between the reaction factors and the response (yield of cetyl octanoate) can be better understood by holding two variable constants and studying the function between the other two variables.

#### 2.2.1. Lipozyme^®^ RMIM

**Figure 2:**
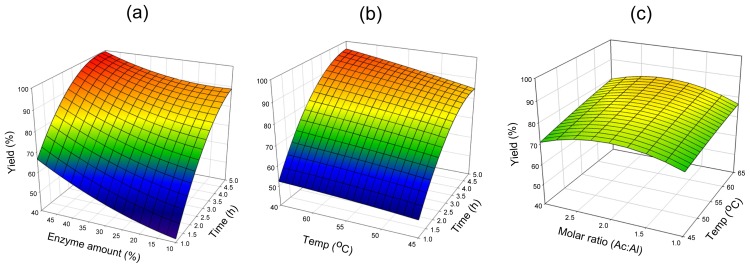
Response surface plots showing the relationships between cetyl octanoate yield and reaction parameters for Lipozyme^®^ RMIM: (**a**) reaction time and enzyme amount; (**b**) reaction time and temperature; (**c**) temperature and substrate molar ratio.

Reaction times and enzyme amount were investigated in the range of the reaction time of 1–5 h and enzyme amount of 10%–50%, respectively. Figure 2a represents the effect of varying reaction time and enzyme amount on esterification efficiency at a substrate molar ratio of 2:1 and a reaction temperature of 55 °C. With the highest reaction time of 5 h, and the highest enzyme amount of 50%, a cetyl octanoate yield of 99% was obtained. Whereas, when the enzyme amount was decreased to 10% and the reaction time shortened to 1 h, only 42% yield remained. This result was consistent with that shown in Table 3, which indicated that both the reaction time and enzyme amount were the two most important parameters. Figure 2b shows the effects of reaction time, temperature and their mutual interaction on cetyl octanoate synthesis at a substrate molar ratio of 2:1 and an enzyme amount of 30%. At any given temperature from 45 °C to 65 °C, an increase in reaction time from 1 to 5 h led to a curvilinear increase in yield from ~50% to ~90%, indicating that the reaction time was one of the important factors in the synthesis of cetyl octanoate. However, the reaction temperature showed an insignificant effect on the yield. The effect of varying reaction temperature and substrate molar ratio on esterification at a reaction time of 3 h and an enzyme amount of 30% is shown in Figure 2c. At any given substrate molar ratio (octanoic acid:cetyl alcohol), the yield was not affected by reaction temperature, the difference in yield was less than 10%. The critical substrate molar ratio can be found around 2:1 (octanoic acid:cetyl alcohol) as shown in Figure 2c. When the substrate molar ratio increased by more than 2:1, a slight decrease in yield occurred. Chowdary *et al.* [27] observed the esterification rate of ethyl hexanoate catalyzed by *Rhizomucor miehei* lipase decreased at high hexanoic acid concentrations. Hari Krishna *et al*. [28] reported a competitive enzyme inhibition by butyric acid during esterification reactions catalyzed by *Rhizomucor miehei* lipase. Our result agreed well with their results. The reason that an increase of substrate molar ratio led to decreases in the yield might be due to inhibition of *Rhizomucor miehei* lipase activity by acidic reaction condition.

#### 2.2.2. Novozym^®^ 435

**Figure 3:**
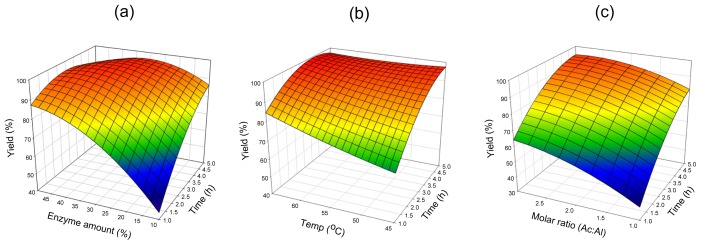
Response surface plots showing the relationships between cetyl octanoate yield and reaction parameters for Novozym^®^ 435: (**a**) reaction time and enzyme amount; (**b**) reaction time and temperature; (**c**) reaction time and substrate molar ratio.

Reaction times and enzyme amount were investigated in the range of the reaction time of 1–5 h and enzyme amount of 10%–50%, respectively. Figure 3a represents the effect of varying reaction time and enzyme amount on esterification efficiency at a substrate molar ratio of 2:1 and a reaction temperature of 55 °C. At the lowest reaction time of 1 h with the lowest enzyme amount of 10%, molar conversion was only 44%. A reaction with an enzyme amount of 35% and a reaction time of 3.75 h increased the yield to 97%. Figure 3b shows the effects of reaction time, temperature and their mutual interaction on cetyl octanoate synthesis at a substrate molar ratio of 2:1 and an enzyme amount of 30%. With the highest reaction temperature of 65 °C and reaction time of 3.75 h, a cetyl octanoate yield of 99% was obtained. Whereas, when the reaction temperature was decreased to 45 °C and the reaction time shortened to 1 h, only 66% yield remained. Figure 3c shows the effect of varying reaction time and substrate molar ratio on esterification at a reaction temperature of 50 °C and an enzyme amount of 20%. The yield increased with increasing substrate molar ratio and reaction time. With the highest reaction time of 5 h and the highest substrate molar ratio of 3:1, cetyl octanoate yield of 95% was obtained. These results from Figure 3 reveal that reaction time, temperature, substrate molar ratio and enzyme amount are all important variables for synthesis of cetyl octanoate catalyzed by *Candida antarctica* (Novozym^®^ 435). Unlike Lipozyme^®^ RMIM, the esterification yield increased as the substrate molar ratio increased using Novozym^®^ 435. Since excess acid in the reaction might cause denaturation of the enzyme and loss of its activity [29], our results indicated that the Novozym^®^ 435 was more tolerant to acidic conditions than Lipozyme^®^ RMIM.

### 2.3. Attaining Optimum Conditions

The optimal point for synthesis of cetyl octanoate was determined by ridge-max analysis, which approximates the estimated ridge of maximum response for increasing radius from the center of the original design. The optimal conditions obtained from the ridge-max analysis are listed in Table 4. The ridge-max analysis predicted maximum yields of Lipozyme^®^ RMIM and Novozym^®^ 435 were 97.56% ± 3.61% and 98.39% ± 1.61%, respectively. The validity of the predicted model was examined by experiments at the predicted optimum conditions. The actual experiment yields were 94.21% ± 1.56% and 98.24% ± 0.11% for Lipozyme^®^ RMIM and Novozym^®^ 435, respectively. The results indicated that the observed values were almost the same as the values predicted from the equation (Equations 1 and 2). Both immobilized lipases can achieve ~95% yield under optimal conditions. However, Novozym^®^ 435 can achieve this yield in a shorter reaction time with a smaller enzyme amount. Salis *et al*. [6] reported that sn-1,3-specific lipase Lipozyme^®^ RMIM was more active than non-specific lipase Novozym^®^ 435 towards wax ester production via transesterification, but Yadav *et al*. [30] found that Novozym^®^ 435 was more active in esterification of levulinic acid with *n*-butanol. As our results suggested, the non-specific lipase Novozym^®^ 435 exhibited less steric hindrance for shorter chain organic acids as compared to other lipases, and showed higher activity in direct esterification.

The ability of Lipozyme^®^ RMIM and Novozym^®^ 435 reuse for cetyl octanoate synthesis under optimum conditions was understood by conducting experiments. After four reaction cycles, the yields decreased from 94% to 80% and 98% to 84% for Lipozyme^®^ RMIM and Novozym^®^ 435, respectively. The results indicated the feasibility of both enzymes recycling in this system. Reuse of catalysts allows expensive enzymes to decrease the process costs of industrial applications.

## 3. Experimental Section

### 3.1. Materials

Lipozyme^®^ RMIM (Particle size 0.2~0.6 mm; 5–6 BAUN/g; Batch acidolysis unit Novo) from *Rhizomucor miehei* (EC 3.1.1.3), supported on a macroporous weak anionic resin, and Immobilized lipase Novozym^®^ 435 (Particle size 0.3~0.9 mm; 10,000 PLU/g; Propyl laurate units) from *Candida antarctica* B (EC 3.1.1.3) were purchased from Novo Nordisk Bioindustrials Inc. (Copenhagen, Denmark). Octanoic acid (99% purity), cetyl alcohol (99% purity) and *n*-hexane were purchased from Sigma Chemical Co. (St Louis, MO, USA). Molecular sieve 4 Å was purchased from Davison Chemical (Baltimore, MD, USA). All other chemicals were purchased from Sigma-Aldrich (St. Louis, MO) and of analytical reagent grade.

### 3.2. Experimental Design

A 5-level-4-factor CCRD was employed in this study, requiring 27 experiments. The fractional factorial design consisted of 16 factorial points, 8 axial points, and 3 center points. The variables and their levels were reaction time (1–5 h), reaction temperature (45–65 °C), substrate molar ratio (Octanoic acid:cetyl alcohol = 1–3:1), and enzyme amount (Lipozyme^®^ RMIM or Novozym^®^ 435/cetyl alcohol = 10%–50%, *w*/*w*). Table 1 shows the independent factors (*X_i_*), levels and experimental design in terms of code and uncode. The 27 runs were performed in a fully random order to avoid bias.

### 3.3. Enzymatic Esterification

All reagents were dehydrated by molecular sieves (4 Å) for 24 h. Cetyl alcohol (0.1 M), different molar ratios of octanoic acid, and different amounts of Lipozyme^®^ RMIM or Novozym^®^ 435, were well mixed in 3 mL *n*-hexane. The esterification reaction was carried out in an orbital shaking water bath (200 rpm) under various reaction temperatures and reaction times as shown in Table 1. The yield % was defined as (mmol of cetyl octanoate/mmol of initial cetyl alcohol) × 100% and was determined using GC method [15].

### 3.4. Statistical Analysis

The experimental data (Table 1) were analyzed by the response surface regression (RSREG) procedure of SAS software (SAS Institute, Cary, NC, USA) to fit the following second-order polynomial equation:

(3) $$Y=b_{k0\text{B}}+\sum_{i=1}^{4}b_{ki}x_{i}+\sum_{i=1}^{4}b_{kii}{x_{i}}^{2}+\sum_{i=1}^{3}\sum_{j=i+1}^{4}b_{kij}x_{i}x_{j}$$

where *Y* is the response (yield %), *B_k0_*, *B_ki_*, *B_kii_* and *B_kij_* are constant coefficients, *x_i_* and *x*_j_ are the uncoded independent variable. The ridge max option in SAS software was used to compute the estimated ridge of maximum response for increasing radius from the center of the original design.

## 4. Conclusions

Optimal systems for producing wax esters by direct esterification of cetyl alcohol with octanoic acid using immobilized lipase (Lipozyme^®^ RMIM and Novozym^®^ 435) in *n*-hexane were developed. RSM and 5-level-4-factor CCRD were employed for optimization of esterification reactions. The maximum yields of cetyl octanoate under the optimal conditions were 94.21% ± 1.56% and 98.24% ± 0.11% for Lipozyme^®^ RMIM and Novozym^®^ 435, respectively. Both enzymes can obtain a high yield of cetyl octanoate, but Novozym^®^ 435 completes the esterification reaction in a shorter reaction time with a smaller amount of enzyme. The RSM studies on the effect of operating variables on wax ester yield will further facilitate the economic optimization of the process before possible scaling up to industrial production.

## Figures and Tables

**Table 1:** CCRD and experimental data for 5-level-4-factor response surface analysis.

Treatment No. a	Factor				Lipozyme^®^ RMIM yield *Y*_1_ (%)	Novozym^®^ 435 yield *Y*_2_ (%)

	Time *X*_1_ (h)	Temp *X*_2_ (°C)	Molar ratio *X*_3_ (Ac:Al) b	Enzyme Amount *X*_4_ (%) c		
1	1(4) b	−1(50)	1(2.5)	−1(20)	81.32 ± 0.76	92.96 ± 0.21
2	1(4)	−1(50)	−1(1.5)	−1(20)	79.45 ± 4.25	84.93 ± 2.02
3	0(3)	0(55)	0(2.0)	2(50)	90.49 ± 1.06	96.07 ± 0.10
4	1(4)	−1(50)	−1(1.5)	1(40)	88.01 ± 1.89	93.90 ± 1.26
5	0(3)	2(65)	0(2.0)	0(30)	80.22 ± 3.72	94.09 ± 2.40
6	−1(2)	−1(50)	−1(1.5)	1(40)	68.40 ± 0.69	86.83 ± 2.80
7	1(4)	1(60)	1(2.5)	1(40)	94.86 ± 1.90	97.39 ± 0.57
8	−1(2)	−1(50)	1(2.5)	1(40)	76.47 ± 2.02	92.18 ± 0.83
9	0(3)	0(55)	0(2.0)	0(30)	81.71 ± 2.60	92.41 ± 0.66
10	−1(2)	−1(50)	−1(1.5)	−1(20)	67.05 ± 2.26	64.16 ± 0.39
11	2(5)	0(55)	0(2.0)	0(30)	92.11 ± 0.29	95.78 ± 0.09
12	−1(2)	1(60)	−1(1.5)	−1(20)	60.25 ± 1.86	79.20 ± 0.89
13	−2(1)	0(55)	0(2.0)	0(30)	44.62 ± 1.93	70.75 ± 0.47
14	−1(2)	1(60)	1(2.5)	−1(20)	70.44 ± 0.98	85.29 ± 3.01
15	0(3)	0(55)	0(2.0)	−2(10)	72.50 ± 0.46	66.83 ± 1.36
16	1(4)	1(60)	1(2.5)	−1(20)	86.90 ± 1.34	97.22 ± 1.74
17	0(3)	0(55)	−2(1.0)	0(30)	73.67 ± 3.62	78.23 ± 2.24
18	−1(2)	−1(50)	1(2.5)	−1(20)	60.22 ± 0.85	76.64 ± 1.93
19	0(3)	0(55)	2(3.0)	0(30)	70.65 ± 2.68	94.28 ± 0.86
20	0(3)	−2(45)	0(2.0)	0(30)	75.49 ± 0.89	88.77 ± 0.41
21	1(4)	1(60)	−1(1.5)	−1(20)	89.75 ± 0.93	91.62 ± 0.29
22	1(4)	1(60)	−1(1.5)	1(40)	90.58 ± 1.54	95.76 ± 0.63
23	−1(2)	1(60)	−1(1.5)	1(40)	78.56 ± 0.64	88.85 ± 2.20
24	−1(2)	1(60)	1(2.5)	1(40)	76.58 ± 2.86	97.15 ± 0.97
25	0(3)	0(55)	0(2.0)	0(30)	80.65 ± 1.05	92.40 ± 2.06
26	1(4)	−1(50)	1(2.5)	1(40)	91.91 ± 1.28	97.95 ± 0.11
27	0(3)	0(55)	0(2.0)	0(30)	80.08 ± 1.63	93.14 ± 1.47

a The treatments were run in random order;

b (Ac:Al) was the molar ratio of octanoic acid:cetyl alcohol;

c The enzyme amount is the weight percentage of cetyl alcohol. (Lipozyme^®^ RMIM or Novozym^®^ 435/cetyl alcohol, *w*/*w*)

**Table 2:** ANOVA for synthetic variables pertaining to the response of percent yield.

Source	Lipozyme^®^ RMIM		Novozym^®^ 435	

	Sum of squares	*p*-Value	Sum of squares	*p*-Value
Linear	2951.08	<0.0001 *	1907.39	<0.0001 *
Quadratic	233.75	0.0887 b	177.31	0.0222 a
Crossproduct	26.51	0.9713 b	197.06	0.0422 a
Total Model	3211.35	0.0001 a	2281.77	<0.0001 *
Lack of Fit	267.29	0.0252 a	124.00	0.0144 a
Pure Error	1.36		0.36	
Total Error	268.65		124.36	

* Very Significant at *p*-value less than 0.0001;

a Significant at *p*-value less than 0.05;

b Insignificant at *p*-value more than 0.05.

**Table 3:** Analysis of variance for joint test.

Factor	Lipozyme^®^ RMIM		Novozym^®^ 435	

	Sum of squares	*p*-Value	Sum of squares	*p*-Value
Time (*X*_1_)	2542.85	<0.0001 a	928.55	<0.0001 a
Temperature (*X*_2_)	87.83	0.5800	189.73	0.0304 a
Molar ratio (*X*_3_)	63.90	0.7214	340.11	0.0037 a
Enzyme amount (*X*_4_)	503.89	0.0153 a	1045.50	<0.0001 a

a Significant at *p*-value less than 0.05.

**Table 4:** Experimental and predicted values of optimization conditions for cetyl octanoate synthesis.

Item	X_1_ (h)	X_2_ (°C)	X_3_ (Ac:Al) a	X_4_ (%)	Predicted yield (%)	Experimental yield (%)
Lipozyme^®^ RMIM	4.00	57.51	2.12	46.41	97.56 ± 3.61	94.21 ± 1.56
Novozym^®^ 435	3.65	57.84	2.35	34.38	98.39 ± 1.61	98.24 ± 0.11

a (Ac:Al) was the molar ratio of octanoic acid:cetyl alcohol.
